# Detection of candidate biomarkers of prostate cancer progression in serum: a depletion-free 3D LC/MS quantitative proteomics pilot study

**DOI:** 10.1038/bjc.2016.291

**Published:** 2016-09-29

**Authors:** S E T Larkin, H E Johnston, T R Jackson, D G Jamieson, T I Roumeliotis, C I Mockridge, A Michael, A Manousopoulou, E K Papachristou, M D Brown, N W Clarke, H Pandha, C L Aukim-Hastie, M S Cragg, S D Garbis, P A Townsend

**Affiliations:** 1 Cancer Sciences Unit, Southampton General Hospital, University of Southampton, Southampton SO16 6YD, UK; 2 Institute of Cancer Sciences, Manchester Cancer Research Centre, Manchester Academic Health Science Centre, University of Manchester, Wilmslow Road, Manchester M20 4QL, UK; 3 Biorelate, BASE, Greenhey's, Manchester Science Park, Pencroft Way, Manchester M15 6JJ, UK; 4 Institute for Life Sciences, Centre for Proteomic Research, University of Southampton, Southampton SO17 1BJ, UK; 5 Faculty of Health and Medical Sciences, University of Surrey, Guildford, Surrey GU2 7TE, UK; 6 Cancer Research UK Cambridge Institute, University of Cambridge, Cambridge CB2 0RE, UK; 7 Institute of Cancer Sciences, Cancer Research UK Manchester Institute, Paterson Building, Wilmslow Road, Manchester M20 4BX, UK; 8 The Christie NHS Foundation Trust, Manchester M20 4BX, UK; 9 Faculty of Health and Medical Sciences, University of Surrey, Guildford, Surrey GU2 7TE, UK

**Keywords:** prostate cancer, serum, proteomics, biomarkers, PSA, LCMS, iTRAQ

## Abstract

**Background:**

Prostate cancer (PCa) is the most common male cancer in the United Kingdom and we aimed to identify clinically relevant biomarkers corresponding to stage progression of the disease.

**Methods:**

We used enhanced proteomic profiling of PCa progression using iTRAQ 3D LC mass spectrometry on high-quality serum samples to identify biomarkers of PCa.

**Results:**

We identified >1000 proteins. Following specific inclusion/exclusion criteria we targeted seven proteins of which two were validated by ELISA and six potentially interacted forming an ‘interactome' with only a single protein linking each marker. This network also includes accepted cancer markers, such as TNF, STAT3, NF-*κ*B and IL6.

**Conclusions:**

Our linked and interrelated biomarker network highlights the potential utility of six of our seven markers as a panel for diagnosing PCa and, critically, in determining the stage of the disease. Our validation analysis of the MS-identified proteins found that SAA alongside KLK3 may improve categorisation of PCa than by KLK3 alone, and that TSR1, although not significant in this model, might also be a clinically relevant biomarker.

The most common male cancer in the United Kingdom is prostate cancer (PCa), with 47 300 diagnoses in 2013 (CRUK, 2014a) and 10 837 deaths in 2012 (CRUK, 2014b) from the disease. At disease presentation, ∼16% of men in the United States will have locally advanced or metastatic disease, despite PSA screening, and of the remainder, 30–40% will still suffer biochemical recurrence regardless of radical prostatectomy (Brawley, 2012). Once PCa has metastasised, life expectancy is generally <5 years. Conversely, patients presenting with organ-confined disease have a minimal risk of death within 15 years (Brawley, 2012). The US screening programme is thought to have led to >1 million additional men being diagnosed and treated for PCa between 1986 and 2005. However, a worrying observation is that for every 1 death that is averted 20 men are ‘overdiagnosed'. Overdiagnosis is a disturbing problem because of globally acknowledged treatment-associated side effects (Welch and Albertsen, 2009).

In this context it becomes essential to discover modes for improving diagnosis and planning surgical interventions. Novel candidate biomarkers offer potential clinical utility in the more accurate identification of patients with an increased risk of aggressive PCa before invasive treatments.

Proteomic profiling utilising isobaric stable isotope labelling and ultra-performance liquid chromatography linked with high-resolution tandem mass spectrometry (LC-MS) offers extended linear dynamic range in proteome coverage (Zeidan and Townsend, 2008; Zeidan *et al*, 2009a, b; Al-Ruwaili *et al*, 2010) with high analytical precision (Garbis *et al*, 2008, 2011).

Such methodological features are particularly important when it comes to the analysis of serum samples whose protein content spans a wide dynamic range of >12 orders of magnitude with the carrier protein albumin accounting for ∼55% of total protein content by mass (Anderson and Anderson, 2002; Boylan *et al*, 2010; Garbis *et al*, 2011; Rehman *et al*, 2012; Tonack *et al*, 2013). Such high abundance of albumin masks or sequesters the presence of lower abundance proteins. Many serum proteomic methods utilise depletion strategies to remove the high abundance proteins (primarily albumin and immunoglobulins) to simplify the analysis of the proteome, but this results in the concurrent removal of many other lower abundance, potentially valuable, proteins (Yocum *et al*, 2005).

Building on the success of previous methods (Garbis *et al*, 2008; Bouchal *et al*, 2009), we developed a quantitative version of a whole serum analysis approach to investigate gender-mediated factors affecting the obesogenic state in humans (Al-Daghri *et al*, 2014). The aim of our current study was to apply this approach to identify serum biomarkers of PCa progression. Our study hypothesis is that the methodological attributes of the iTRAQ 3D LC-MS protocol exhibits sufficient selectivity, specificity and sensitivity to reveal novel and clinically relevant biomarkers that can stage PCa progression.

## Materials and methods

### Discovery samples

For the mass spectrometry (MS) discovery phase, we used serum from a panel of patients recruited (using informed consent) through the University of Surrey (Professor Pandha SUN study, REC reference 08/H1306/115) categorised as follows: (1) PCa null, <1 ng ml^−1^ PSA (20 samples in this category); (2) putative benign disease, 4.7–12 ng ml^−1^ PSA, including benign prostatic hyperplasia, prostatitis, prostatic intraepithelial neoplasia, inflammation and atrophy (15 samples in this category); (3) T1–T2 stage prostate cancer, 3.9–4.8 ng ml^−1^ PSA (20 samples in this category); and (4) T3–T4 stage PCa (some with metastatic disease), 6.7–17.65 ng ml^−1^ PSA (20 samples in this category). Serum was collected in red-topped serum activator tubes (BD Biosciences, Oxford, UK), inverted five times and left at room temperature for 30 min before centrifugation at 3000 r.p.m. for 10 min. All samples were centrifuged within 2 h of collection. After centrifugation, the top clear fraction (serum) was removed and aliquoted into cryovials (1 ml per vial) before being stored at −80 °C.

### Validation samples

To validate biomarkers by ELISA, we used a separate, independent cohort of samples collected through the University of Manchester (Professor Noel Clarke, Northern Prostate Cancer Collaborative (ProMPT), MREC/01/4/061). These samples were categorised as follows: (1) PCa null (20 samples); (2) patients with BPH (20 samples); (3) T1–T2 stage PCa, 0.7–31 ng ml^−1^ PSA (20 samples); and (4) T3–T4 stage PCa (some with metastatic disease), 0.5–1400 ng ml^−1^ PSA (20 samples). Blood was collected in Gold-topped BD Vacutainer SST II Plus plastic serum tube (BD Biosciences 367955), inverted five times and left at room temperature for a minimum of 30 min (up to 2 h) before centrifugation at 1000 **g** for 10 min. Serum was removed and aliquoted before storing at −80 °C.

### LC-MS proteomics

**Figure 1:**
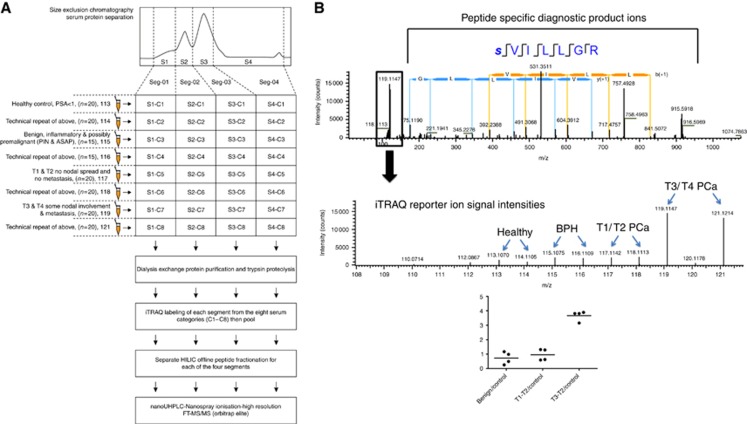
**Mass Spectrometry Methodology**. (**A**) Illustration of the multiplex quantitative serum proteomics method used for the discovery findings. TR=technical repeat, iTRAQ labels are in bold. The method utilises multidimensional liquid chromatography, stable isotope labelling of surrogate tryptic peptides and ultra-high resolution/precision tandem mass spectrometry using the state-of-the-art FT-Obritrap Elite platform. (**B**) Annotated high-resolution (FTMS) product ion mass spectrum of the tryptic peptide SVILLGR, uniquely traceable to PSA with an expanded view of the low-mass region (and a dot plot) showing the observed iTRAQ reporter ion intensities, demonstrating the highest abundance of the PSA proteotypic peptide occurring for the T3–T4 PCa clinical cohorts.

All aspects of the LC-MS proteomics method used for this study have been reported by the authors (Al-Daghri *et al*, 2014). The offline HILIC peptide separation has also been reported by the authors (Garbis *et al*, 2011; Delehouze *et al*, 2014; Bouchal *et al*, 2015). The discovery experiment was executed once. However, technical replicates of each group were performed using the same samples. The samples were pooled twice and labelled differently to provide these technical repeats (Figure 1A). Each pooled serum category was analysed in parallel under the same offline tryptic peptide LC-MS conditions. This gave us a high degree of analytical precision and the ability to more reliably determine a smaller degree of differential analysis not feasible with label-free methods. The biological and technical reproducibility of the study method has been reported by the authors (Al-Daghri *et al*, 2014). Specific method details may be found in the Supplementary Methods section. The mass spectrometric proteomics data have been deposited to the ProteomeXchange Consortium (Vizcaino *et al*, 2014) via the PRIDE partner repository (Wang *et al*, 2012; Vizcaino *et al*, 2014; Vizcaino *et al*, 2013) with the data set identifier PXD004575.

**Figure 2:**
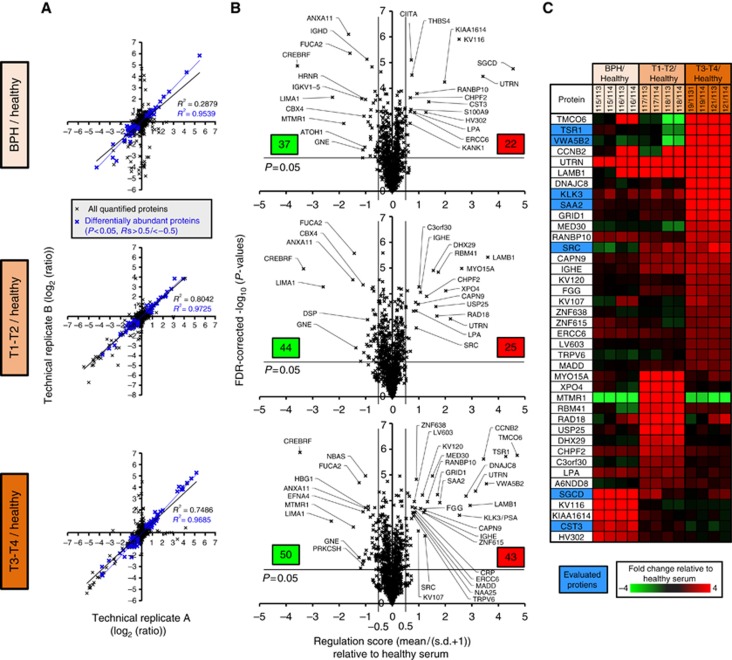
**Summary of the reproducible differential serum protein abundance observed in BPH, T1–T2 and T3–T4 relative to healthy serum.**(**A**) The reproducibility between technical replicates for all proteins, highlighting those considered differentially abundant (blue), relative to healthy serum (labels 113 and 114). (**B**) Volcano plots highlighting significantly, differentially abundant proteins plotting regulation scores (Rs) and −log_10_(*P*-values) of the four ratios derived from the technical/biological replicates for BPH, T1–T2 and T3–T4 relative to healthy donor serum. A total of 72 and 82 proteins demonstrated significant differential abundance (Rs>0.5 or Rs<−0.5, *P*<0.05) in at least one of the three conditions, respectively, totalling 151 distinct differentially abundant proteins. (**C**) The top 40 significantly (*P*<0.05) overabundant proteins, sorted by regulation score, across the BPH, T1–T2 and T3–T4 samples relative to healthy serum. Highlighted proteins are the seven selected for further validation by ELISA.

Our protein selection process is depicted in Supplementary Figure S1. For each group studied by MS, there were two technical replicates, labelled with a different iTRAQ label, resulting in four ratios for each comparison (i.e., 115/113, 115/114, 116/113 and 116/114 for BPH/control). Because of the variability observed between replicates, a measure, termed the ‘regulation score' (Equation (1)) was used to summarise both the magnitude and consistency of differential abundance across multiple derived log_2_(ratios). For instance, when the mean is high and the s.d. is low, the resulting regulation score is high. The top 40 most consistently regulated, significant (*P*<0.05) proteins were derived from the regulation score values for the three conditions. This shortlist was used for the selection of validation markers (Figure 2C).


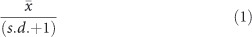


Equation (1) shows the calculation of the regulation score.

From the shortlist we selected proteins that differentiated one disease group from the other two, or had a step-wise increase or decrease with progression, and, critically, had commercially available validation reagents (Table 1). Because of lack of commercial availability, we were unable to study the markers that seemed to be specific to early-stage PCa.

### ELISA validation

The ELISAs were obtained from Antibodies Online and My Biosource, details are shown in Supplementary Table S1. The ELISAs were performed according to the manufacturer's protocols. More detail can be found in the Supplementary Methods section.

### Literature and network analysis

In addition to the ELISA validation of our chosen MS-identified biomarkers, a literature and network analysis of interacting proteins with the seven markers was performed in collaboration with Biorelate (www.biorelate.com). Details can be found in the Supplementary Methods section.

## Results

### Discovery MS

Our LC-MS proteomics method (Figure 1A) enabled us to identify a total of 1034 proteins (Supplementary Table S2). Our raw data have been uploaded to the PRIDE database (Accession: PXD004575). As a proof of principle, our method allowed the non-targeted relative quantitative analysis of the low-abundant KLK3 (PSA) protein without the need for mainstream immunodepletion strategies that may have otherwise depleted it (Figure 1B). Our KLK3 finding demonstrates its well-documented limitation to discriminate BPH from early-stage PCa at the serum level, but does demonstrate its utility as a recurrence marker because of the high levels seen in later-stage disease.

As a means to assess the absolute abundance range of our quantified proteome we compared the total number of peptide-spectrum matches (PSMs) for each protein across all four segments with published and estimated concentration data from PeptideAtlas (Farrah *et al*, 2011). We found a linear relationship between our MS-based average counts and the absolute concentrations for >350 proteins, suggesting that approximate absolute abundances for previously unidentified proteins can be predicted by the PSM counts (Supplementary Figure S2).

To identify functional associations between the differentially expressed proteins we created a protein–protein interaction network using the Genes2FANs (Dannenfelser *et al*, 2012) tool (Supplementary Figure S3A) that confirmed that a set of regulated proteins is functionally related although not always through direct interactions. Gene Ontology analysis (Chen *et al*, 2013) showed significant enrichment of extracellular vesicular exosome proteins (Supplementary Figure S3B), suggesting a potential secretion route of the differentially expressed proteins into the blood stream.

A total of 1034 serum proteins were identified with excellent reproducibility between technical replicates (Figure 2A). From this total, we shortlisted 40 for further study based on the regulation score mentioned previously. The distribution of regulation scores for the markers is displayed in Figure 2B and the top 40 in Figure 2C. Of these, seven were selected for validation by ELISA (Supplementary Table S3) based on their ability to differentiate one group (control/benign/T1–T2 PCa/T3–T4 PCa) from another (according to the MS data) and the availability of commercial reagents. These seven markers are summarised in Table 1.

### Literature and network analysis

Using all PubMed abstracts and all PubMed Central (PMC) open-access full-text articles, we performed a comprehensive literature analysis of the seven markers to assess their previous relevance with respect to PCa and as biomarkers (Table 2). Only VWA5B2 had not been studied in the context of PCa previously, with just three publications found in total. Research was limited in PCa for both SGCd and TSR1. Of note, Love *et al* (2009) demonstrated SGCd to have a 14-fold increased level of extracellular expression in BPH RNA compared with PCa RNA, whereas Savas *et al* (2010) identified single-nucleotide polymorphisms (SNPs) associated with SGCd and selenium resistance – a dietary trace element shown to protect against various cancers including PCa (Platz and Helzlsouer, 2001; Meuillet *et al*, 2004; Neill and Fleshner, 2006). No evidence was found that supported TSR1 as a PCa biomarker. The remaining four biomarkers CST3, SRC, SAA1 and KLK3, were found to have been intensively studied in PCa biology, with each having ⩾40 PCa biomarker-associated publications. In keeping with our MS data, CST3 has been shown to be downregulated in PCa (Jiborn *et al*, 2006; Wegiel *et al*, 2009). SAA1 has been identified as a marker for distinguishing PCa patients with bone lesions (Le *et al*, 2005). SRC has been shown, as it has with many other cancers, to be upregulated in PCa, with a large resource of research available. The existing use of KLK3 as a gold standard marker for PCa is clearly reflected in the 4908 associated PCa biomarker publications found.

**Figure 3:**
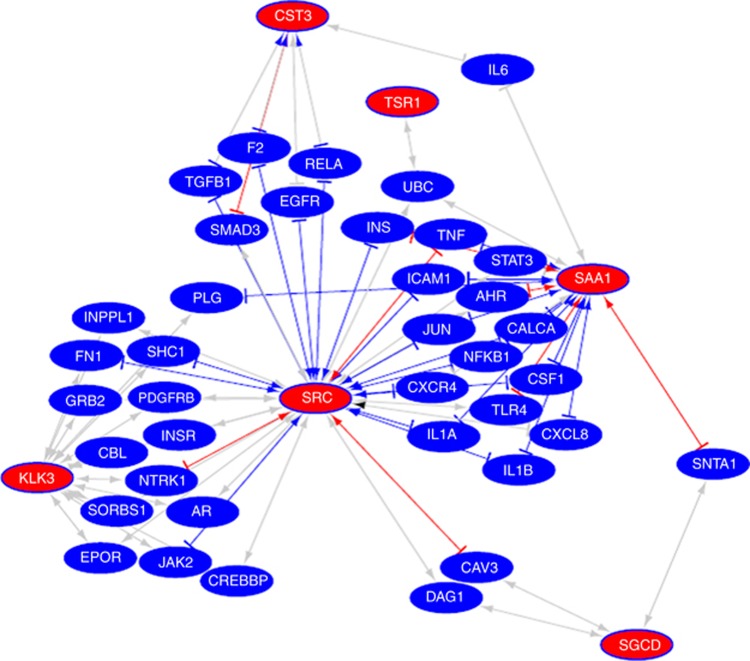
**Network of markers excluding VWA5B2 (for which there were no discernible interactions) and proteins that have been shown to interact (directly or indirectly) with at least two of these, as identified from the publications associated with each marker and from the STRING database.**

Using each biomarker and their associated publications, we then curated any documented interactions (both direct and indirect) with other proteins, supplementing these with any additional interactions stored in the STRING database. As a result, interactions were discerned for all markers except for VWA5B2. These were then combined to form a network of interactions between proteins linking each of the six markers (Figure 3). Interestingly, these form a coherent graph with SRC, SAA1 and KLK3 contributing the most connecting proteins between the markers. An analysis, using DAVID (Huang da *et al*, 2009), of the enriched Gene Ontology (GO) terms associated with the six markers and their connecting interactants revealed positive regulation of biosynthetic process (*P*=5.82E-18), positive regulation of cellular biosynthetic process (*P*=8.64E−17) and positive regulation of multicellular organismal process (*P*=2.81E−16) as the most enriched terms (Table 2). Other significantly enriched GO terms that may be indicative of this group's role in PCa include regulation of cytokine production (*P*=1.78E−13) and regulation of cell migration (*P*=2.55E−12).

### Biomarker validation by ELISA

**Figure 4:**
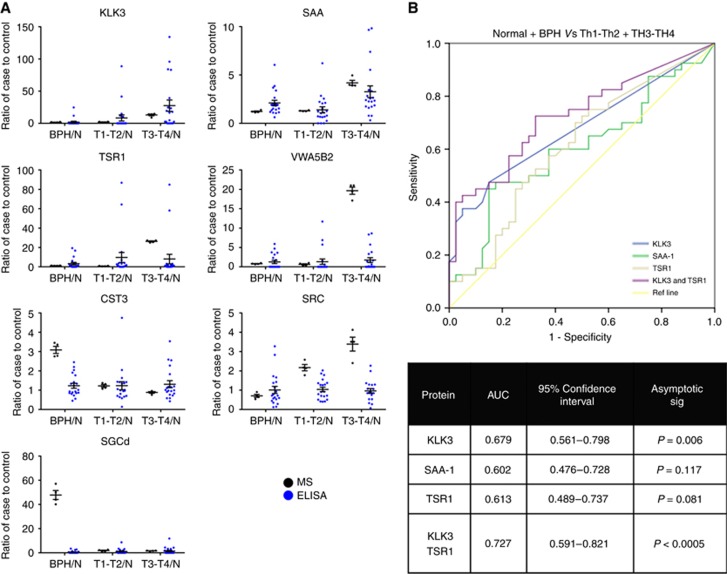
**Biomarker Panel Validation.**(**A**) The MS (black dots) and ELISA (blue dots) data for each marker detected. Dots represent ratios of the individual value for each case in the groups (BPH, T1–T2 and T3–T4) to the mean of the normal group. The abundance of each marker was calculated from a 5PL curve of the intensity values from the ELISAs. (**B**) The ROC curve analysis of individual markers and binary logistic regression model containing KLK3 and TSR1.

The ELISAs, as a clinically accepted diagnostics method, were performed on an independent cohort of patients and results compared with the discovery MS data (Figure 4A). Kruskal–Wallis analysis of the ELISA data demonstrated that SAA and KLK3 (PSA) were significantly differentially expressed across the groups (*P*<0.001). Pairwise Mann–Whitney *U* analysis showed significant SAA increases in levels in benign and T1–T2 PCa (*P*=0.037), benign and control (*P*=0.001), T3–T4 and control (*P*<0.001) and T1–T2 and T3–T4 (*P*=0.002). The KLK3 ELISA concentration was consistent with the MS data, with T3–T4 being significantly different to the control (*P*<0.001), benign (*P*<0.001) and T1–T2 (*P*=0.010) groups. T1–T2 was also found to be significantly different to the control group (*P*=0.009).

TSR1 was found not to be significant by Kruskal–Wallis analysis, but differences were found by pairwise Mann–Whitney *U* with levels increasing in benign *vs* control, T1–T2 *vs* control and T3–T4 *vs* control, although only the T1–T2 *vs* control was significant (*P*=0.013). TSR1 was identified as a T3–T4 stage PCa marker according to MS, yet the ELISA data suggest it to be a marker of ‘cellular change' as it was significantly increased in benign and the two PCa groups compared with the control group, but not differentially expressed between the three disease groups.

Using ELISA, SGCd, SRC, CST3 and VWA5B2 did not show any significant differences in abundance across the disease groups. The ELISA validation was technically difficult as the data imply that the levels of the target proteins largely fall below the detection limits of such assays.

To provide further insight into the utility of these markers, we performed binary logistic regression to produce predictive models that were then analysed by ROC curves (Figure 4B). This analysis showed that KLK3 had an AUC of 0.679 significantly different from the null hypothesis of AUC=0.5 (*P*=0.006). Both SAA-1 and TSR1 had AUC values of 0.602 and 0.613, respectively. Alone these markers were not considered significantly different from the null hypotheses. However, using TSR1 in combination with KLK3 gave an AUC value of 0.727, improving on the predictability of KLK3 alone that is significantly different from the null hypothesis (*P*<0.0005).

## Discussion

The iTRAQ 3D-LC-MS analysis of pooled serum samples yielded many putative targets for validation. Of note, KLK3 (PSA) was identified, and its abundance across the groups was in keeping with current literature and clinical experience. High levels were observed in late-stage PCa but fairly similar levels were found in the control and benign disease groups, with a slight increase in the early-stage PCa group (not significant). There are few other MS studies that have managed to identify KLK3 in serum samples from PCa patients, probably because of the use of immunodepletion strategies used in those studies (Adam *et al*, 2002; Rehman *et al*, 2012). To overcome this, studies have been utilising immunoprecipitation (IP) MS to ‘extract' PSA for MS analysis. Utilising stable isotope labelling-multiple reaction monitoring MS (SIL/MRM-MS), it has been possible for one group to simultaneously measure multiple biomarkers including various PSA forms (Chen *et al*, 2015).

We chose several potential biomarkers identified by iTRAQ 3D-LC-MS analysis for further analysis in an attempt to find biomarkers that together might allow the improved prediction of PCa stage. These ELISAs were used to investigate the abundance of these proteins in individual patient samples. Ideally, the panel would include proteins that showed different patterns of abundance between groups to allow additional predictability. The panel thus included proteins that should increase abundance in a single patient group as well as proteins that showed progressive changes across patient groups. Availability of ELISAs for the early-stage PCa markers was limited. An ELISA kit for USP24 was obtained, but was unable to detect the marker in serum (data not shown) and hence this was excluded from further analysis. It was noted that the Cancer Genome Atlas Research Network (2015) identified an amplification of a region that includes XPO4 (13q12.11), one of the markers identified in our MS discovery phase as a putative marker of early-stage disease.

Both SAA and KLK3 were found to be significantly differentiated by Kruskal–Wallis analysis of the ELISA data with very significant agreement between the two diagnostic methods. It is unsurprising that there was a lack of further validated markers as the stability of serum proteins can be poor (Gislefoss *et al*, 2009). For example, studies of KLK3 (PSA) stability advise caution in any analyses done on serum KLK3 after 2 years of storage at −70 °C (Woodrum and York, 1998). As MS, by its very nature, studies proteins by looking at peptide signatures of proteins, it is less hindered by this degradation than ELISA for whole proteins would be.

Our literature analysis was useful in providing an improved rigorous understanding of PCa with respect to each associated marker. We found that although TSR1 has not been strongly associated with PCa previously, it has been suggested to play a putative role in the quality control of 18S rRNA precursor production (Tafforeau *et al*, 2013). Taking this alongside the importance of ribosome biogenesis in cancer (van Sluis and McStay, 2014), it is perhaps unsurprising that a molecule involved in this process has implicated in PCa. In addition, work on tissue has shown TSR1 RNA expression in prostate tissue, although its highest levels of expression are found in the testis (Ardlie *et al*, 2015).

SRC is a non-receptor protein tyrosine kinase that has a number of roles in cell signalling (Wheeler *et al*, 2009). These interactions are thought to lead to several functions such as proliferation, growth differentiation, motility, migration, angiogenesis and survival. Hence, it has been implicated in several cancers including PCa as it underpins many of the hallmarks of cancer as described by Hanahan and Weinberg (2011). Previous studies utilising the SRC inhibitor dasatinib in PCa cell lines suggests that SRC may be a mediator of cell growth and migration (Nam *et al*, 2005). The PCa clinical trials with dasatinib (SRC family kinase inhibitor) have been promising, with a reduction in bone resorption in over half of the patients with progressive metastatic prostate cancer (Wheeler *et al*, 2009), where bone is the prime metastatic site for PCa. Interestingly, the GTEx project database listed prostate as the highest SRC RNA expressing tissue (Ardlie *et al*, 2015).

CST3 has been shown to be downregulated in PCa and is thought to have a role in invasion through the MAPK/ERK and androgen receptor pathways (Wegiel *et al*, 2009). A role for CST3 in neuroendocrine differentiation in PCa has also been suggested (Jiborn *et al*, 2006). Here, CST3 was downregulated in non-neuroendocrine tumour tissue and that this downregulation correlated with increasing Gleason Grade. In PCa neuroendocrine tumours (a highly aggressive subtype), however, the abundance increased with Gleason grade (Jiborn *et al*, 2006). CST3 belongs to a family of cysteine protease inhibitors that prevent proteolysis of, for example, the extracellular matrix and basement membrane. Downregulation of these inhibitors (and dysregulation of the proteolytic/anti-proteolytic homeostasis) are associated with malignant progression and shorter mean patient survival (Jiborn *et al*, 2006). Imbalance in these molecules can occur in response to inflammatory diseases that could potentially account for its upregulation in the benign disease group that includes conditions such as prostatitis. CST3 RNA seems to be expressed fairly ubiquitously with higher expression seen in the brain, although expression has been observed in prostate tissue (Ardlie *et al*, 2015), and particularly in PCa (Uhlén *et al*, 2015).

SAA was identified from MS as being more abundant in late-stage disease that was supported by ELISA data. Le *et al* (2005) identified SAA as a marker in PCa patients showing increased levels in serum to be indicative of the presence of bone metastasis. SAA is an acute-phase protein associated with inflammation, and hence it is unlikely to be PCa specific but, in conjunction with other PCa biomarkers, could be a useful addition to a panel of (companion) biomarkers.

A limitation to the iTRAQ 3D LC-MS analysis used for our study was the use of pooled specimens for each clinical cohort. Essential to the pooled clinical cohorts was the implementation of our well-defined inclusion and exclusion criteria that minimised confounding factors. Ideally, the proteomic analysis of individual, non-pooled specimens would have allowed the assessment of heterogeneity between individual samples. The lack of validation of some of our candidate markers could in part be related to the heterogeneity of PCa itself and the variability between the two cohorts. Prostate cancer is renowned for its clinical heterogeneity in terms of treatment response, speed of growth and overall prognosis, but it is also an incredibly complex disease at the molecular level (Boyd *et al*, 2012). This molecular heterogeneity may account for the difficulty of identifying commonalities with pooled samples, and also for the low validation rate seen between our discovery and validation cohorts that were taken from distinct geographical UK locations.

The inability of ELISA to validate some of the MS identified biomarkers may also be attributed to fundamental differences between the LC-MS approach and the ELISA technique. ELISAs rely upon an intact interaction between an epitope and antigen and are thus dependent on both the integrity of analyte and quality of the antibody. Conversely, MS is not limited by these factors and, in fact, is reliant upon the detection and identification of peptide fragments and hence is less hampered by epitope degradation. The gold standard in verifying the absolute quantitative accuracy of our proposed biomarkers is the use of targeted LC-MS approaches using such tandem mass spectrometry techniques as multiple reaction monitoring (MRM), parallel reaction monitoring (PRM) or selected reaction monitoring (SRM). To increase their sensitivity the LC-MS technique can be combined with affinity capture and purification of target proteins or their surrogate peptides, as reported in the literature (Boja and Rodriguez, 2012) However, such LC-MS-based approaches require considerable method development and were beyond the scope of this proof-of-concept biomarker discovery study. We chose the ELISA assay as a low-cost alternative that is in wide commercial use for protein measurements.

Despite difficulties with validating potential biomarkers and a relatively small sample size, we identified SAA and TSR1 as biomarkers that could potentially add to the predictability of KLK3 and successfully validated these via ELISA. When analysed using ROC curves, TSR1 in particular was able to add to the predictability of KLK3, increasing the AUC from 0.679 to 0.737. SAA did little to increase the ability of KLK3 to distinguish between cancer and noncancer, but pairwise Mann–Whitney *U* analysis suggested it may have a role in distinguishing different stages of cancer and should not be dismissed as a potentially useful biomarker in a future biomarker panel.

In conclusion, as a proof-of-principle study, our serum proteomics discovery pipeline allows the discovery of novel serological markers of PCa progression of potential clinical utility. Our analysis has identified two potential biomarkers, SAA and TSR1, that could be combined with KLK3 to improve its predictive capability of disease progression. These proposed biomarkers warrant validation across hundreds of samples in a blinded randomised control setting. Such a validation process must also include well-curated serum specimens derived from diverse populations with well-defined patient information (BMI, family history, pharmacological status, etc.). The validation of the proposed biomarker panel constitutes a future perspective and is beyond the scope of this proof-of-concept study.

## Figures and Tables

**Table 1:** Summary data of the seven proteins identified via mass spectrometry that were shortlisted for ELISA validation

**Protein**	**log2 benign/control**				**log2 T1–T2/control**				**log2 T3–T4/control**			
Delta-sarcoglycan	5.329	5.457	5.643	5.837	1.159	1.385	0.232	0.377	−0.021	0.104	0.699	0.846
Pre-rRNA-processing protein TSR1 homologue	0.334	0.452	0	0.078	−0.058	0.014	−1.11	−1.012	4.555	4.676	4.642	4.77
Kalikrein 3	0.498	1.174	0.261	0.988	0.633	1.32	0.596	1.295	3.204	3.892	3.161	3.818
von Willebrand factor A domain-containing protein 5B2	−0.312	−0.026	−0.682	−0.435	−0.253	−0.012	−1.836	−1.569	3.991	4.28	4.096	4.393
Serum amyloid A protein	0.471	0.288	0.181	0.209	0.379	0.449	0.325	0.327	2.046	1.992	2.309	1.965
Proto-oncogene tyrosine-protein kinase Src	−0.633	−0.879	−0.151	−0.437	1.118	0.827	1.366	1.1	1.506	1.263	2.044	1.809
Cystatin-C	1.476	1.749	1.461	1.785	0.172	0.456	0.097	0.394	−0.345	−0.06	−0.405	−0.15

Abbreviation: ELISA=enzyme-linked immunosorbent assay.

These were selected based on their ability to differentiate one patient group from another (for example, delta-sarcoglycan (SGCd) showed marked overexpression in benign prostatic hyperplasia (BPH) compared with control, but T1–T2 and T3–T4 were similar to the control) or their stepwise increase over the course of the disease (serum amyloid A (SAA)) and the availability of commercial reagents.

**Table 2:** Literature-informatic analysis for PCa marker proteins

**Marker**	**Total publications**	**PCa publications**	**PCa publications enrichment** ***P-*****value**	**PCa biomarker publications**	**PCa biomarker publications enrichment** ***P-*****value**
SGCD	323	3	0.60	2	0.90
TSR1	112	2	0.29	0	1.00
VWA5B2	3	0	1.00	0	1.00
CST3	4431	60	5.85E−3	50	1.00
SRC	7805	618	5.28E−342	305	1.19E−1201
SAA1	3767	58	4.21E−4	48	1.00
KLK3	44 017	19 295	1.75E−25 391	4908	1.92E−3105

Abbreviation: PCa=prostate cancer.

Publications were assigned to each protein if they were mentioned in the text. Prostate cancer (PCa) publications are those marker publications filtered that also mention PCa-related terms. The PCa biomarker publications are those PCa publications further filtered for mentions of biomarker-related terms. Enrichment *P*-values were calculated using Fisher's exact test (see Materials and Methods).

## References

[bib1] Adam B-L, Qu Y, Davis JW, Ward MD, Clements MA, Cazares LH, Semmes OJ, Schellhammer PF, Yasui Y, Feng Z, Wright GL (2002) Serum protein fingerprinting coupled with a pattern-matching algorithm distinguishes prostate cancer from benign prostate hyperplasia and healthy men. Cancer Res 62(13): 3609–3614. 12097261

[bib2] Al-Daghri NM, Al-Attas OS, Johnston HE, Singhania A, Alokail MS, Alkharfy KM, Abd-Alrahman SH, Sabico SL, Roumeliotis TI, Manousopoulou-Garbis A, Townsend PA, Woelk CH, Chrousos GP, Garbis SD (2014) Whole serum 3D LC-nESI-FTMS quantitative proteomics reveals sexual dimorphism in the milieu interieur of overweight and obese adults. J Proteome Res 13(11): 5094–5105. 25072778 10.1021/pr5003406

[bib3] Al-Ruwaili JA, Larkin SE, Zeidan BA, Taylor MG, Adra CN, Aukim-Hastie CL, Townsend PA (2010) Discovery of serum protein biomarkers for prostate cancer progression by proteomic analysis. Cancer Genomics Proteomics 7(2): 93–103. 20335524

[bib4] Anderson NL, Anderson NG (2002) The human plasma proteome history, character, and diagnostic prospects. Mol Cell Proteomics 1(11): 845–867. 12488461 10.1074/mcp.r200007-mcp200

[bib5] Ardlie KG, Deluca DS, Segrè AV, Sullivan TJ, Young TR, Gelfand ET, Trowbridge CA, Maller JB, Tukiainen T, Lek M, Ward LD, Kheradpour P, Iriarte B, Meng Y, Palmer CD, Esko T, Winckler W, Hirschhorn JN, Kellis M, MacArthur DG, Getz G, Shabalin AA, Li G, Zhou Y-H, Nobel AB, Rusyn I, Wright FA, Lappalainen T, Ferreira PG, Ongen H, Rivas MA, Battle A, Mostafavi S, Monlong J, Sammeth M, Mele M, Reverter F, Goldmann JM, Koller D, Guigó R, McCarthy MI, Dermitzakis ET, Gamazon ER, Im HK, Konkashbaev A, Nicolae DL, Cox NJ, Flutre T, Wen X, Stephens M, Pritchard JK, Tu Z, Zhang B, Huang T, Long Q, Lin L, Yang J, Zhu J, Liu J, Brown A, Mestichelli B, Tidwell D, Lo E, Salvatore M, Shad S, Thomas JA, Lonsdale JT, Moser MT, Gillard BM, Karasik E, Ramsey K, Choi C, Foster BA, Syron J, Fleming J, Magazine H, Hasz R, Walters GD, Bridge JP, Miklos M, Sullivan S, Barker LK, Traino HM, Mosavel M, Siminoff LA, Valley DR, Rohrer DC, Jewell SD, Branton PA, Sobin LH, Barcus M, Qi L, McLean J, Hariharan P, Um KS, Wu S, Tabor D, Shive C, Smith AM, Buia SA, Undale AH, Robinson KL, Roche N, Valentino KM, Britton A, Burges R, Bradbury D, Hambright KW, Seleski J, Korzeniewski GE, Erickson K, Marcus Y, Tejada J, Taherian M, Lu C, Basile M, Mash DC, Volpi S, Struewing JP, Temple GF, Boyer J, Colantuoni D, Little R, Koester S, Carithers LJ, Moore HM, Guan P, Compton C, Sawyer SJ, Demchok JP, Vaught JB, Rabiner CA, Lockhart NC, Ardlie KG, Getz G, Wright FA, Kellis M, Volpi S, Dermitzakis ET (2015) The Genotype-Tissue Expression (GTEx) pilot analysis: Multitissue gene regulation in humans. Science 348(6235): 648–660. 25954001 10.1126/science.1262110 PMC4547484

[bib6] Boja ES, Rodriguez H (2012) Mass spectrometry-based targeted quantitative proteomics: achieving sensitive and reproducible detection of proteins. Proteomics 12(8): 1093–1110. 22577011 10.1002/pmic.201100387

[bib7] Bouchal P, Dvorakova M, Roumeliotis T, Bortlicek Z, Ihnatova I, Prochazkova I, Ho JT, Maryas J, Imrichova H, Budinska E, Vyzula R, Garbis SD, Vojtesek B, Nenutil R (2015) Combined proteomics and transcriptomics identifies carboxypeptidase B1 and nuclear factor kappaB (NF-kappaB) associated proteins as putative biomarkers of metastasis in low grade breast cancer. Mol Cell Proteomics 14(7): 1814–1830. 25903579 10.1074/mcp.M114.041335 PMC4587321

[bib8] Bouchal P, Roumeliotis T, Hrstka R, Nenutil R, Vojtesek B, Garbis SD (2009) Biomarker discovery in low-grade breast cancer using isobaric stable isotope tags and two-dimensional liquid chromatography-tandem mass spectrometry (iTRAQ-2DLC-MS/MS) based quantitative proteomic analysis. J Proteome Res 8(1): 362–373. 19053527 10.1021/pr800622b

[bib9] Boyd LK, Mao X, Lu Y-J (2012) The complexity of prostate cancer: genomic alterations and heterogeneity. Nat Rev Urol 9(11): 652–664. 23132303 10.1038/nrurol.2012.185

[bib10] Boylan KL, Andersen JD, Anderson LB, Higgins L, Skubitz AP (2010) Quantitative proteomic analysis by iTRAQ for the identification of candidate biomarkers in ovarian cancer serum. Proteome Sci 8: 31. 20546617 10.1186/1477-5956-8-31 PMC2893134

[bib11] Brawley OW (2012) Trends in prostate cancer in the United States. J Natl Cancer Inst Monogr 2012(45): 152–156. 23271766 10.1093/jncimonographs/lgs035 PMC3540881

[bib12] Cancer Genome Atlas Research Network (2015) The molecular taxonomy of primary prostate cancer. Cell 163(4): 1011–1025. 26544944 10.1016/j.cell.2015.10.025 PMC4695400

[bib13] Chen EY, Tan CM, Kou Y, Duan Q, Wang Z, Meirelles GV, Clark NR, Ma'ayan A (2013) Enrichr: interactive and collaborative HTML5 gene list enrichment analysis tool. BMC Bioinformatics 14: 128. 23586463 10.1186/1471-2105-14-128 PMC3637064

[bib14] Chen Y-T, Tuan L-P, Chen H-W, Wei IA, Chou M-Y, Chen H-M, Tyan Y-C, Chen S-F (2015) Quantitative analysis of prostate specific antigen isoforms using immunoprecipitation and stable isotope labeling mass spectrometry. Anal Chem 87(1): 545–553. 25427836 10.1021/ac5033066

[bib15] CRUK (2014a) Prostate cancer incidence statistics for the UK. Vol. 2015.

[bib16] CRUK (2014b) Prostate cancer mortality statistics for the UK. Vol. 2015.

[bib17] Dannenfelser R, Clark NR, Ma'ayan A (2012) Genes2FANs: connecting genes through functional association networks. BMC Bioinformatics 13: 156. 22748121 10.1186/1471-2105-13-156 PMC3472228

[bib18] Delehouze C, Godl K, Loaec N, Bruyere C, Desban N, Oumata N, Galons H, Roumeliotis TI, Giannopoulou EG, Grenet J, Twitchell D, Lahti J, Mouchet N, Galibert MD, Garbis SD, Meijer L (2014) CDK/CK1 inhibitors roscovitine and CR8 downregulate amplified MYCN in neuroblastoma cells. Oncogene 33(50): 5675–5687. 24317512 10.1038/onc.2013.513 PMC4087096

[bib19] Farrah T, Deutsch EW, Omenn GS, Campbell DS, Sun Z, Bletz JA, Mallick P, Katz JE, Malmstrom J, Ossola R, Watts JD, Lin B, Zhang H, Moritz RL, Aebersold R (2011) A high-confidence human plasma proteome reference set with estimated concentrations in PeptideAtlas. Mol Cell Proteomics 10(9): M110 006353. 10.1074/mcp.M110.006353 PMC3186192 21632744

[bib20] Garbis SD, Roumeliotis TI, Tyritzis SI, Zorpas KM, Pavlakis K, Constantinides CA (2011) A novel multidimensional protein identification technology approach combining protein size exclusion prefractionation, peptide zwitterion-ion hydrophilic interaction chromatography, and nano-ultraperformance RP chromatography/nESI-MS2 for the in-depth analysis of the serum proteome and phosphoproteome: application to clinical sera derived from humans with benign prostate hyperplasia. Anal Chem 83(3): 708–718. 21174401 10.1021/ac102075d

[bib21] Garbis SD, Tyritzis SI, Roumeliotis T, Zerefos P, Giannopoulou EG, Vlahou A, Kossida S, Diaz J, Vourekas S, Tamvakopoulos C, Pavlakis K, Sanoudou D, Constantinides CA (2008) Search for potential markers for prostate cancer diagnosis, prognosis and treatment in clinical tissue specimens using amine-specific isobaric tagging (iTRAQ) with two-dimensional liquid chromatography and tandem mass spectrometry. J Proteome Res 7(8): 3146–3158. 18553995 10.1021/pr800060r

[bib22] Gislefoss RE, Grimsrud TK, Morkrid L (2009) Stability of selected serum proteins after long-term storage in the Janus Serum Bank. Clin Chem Lab Med 47(5): 596–603. 19290843 10.1515/CCLM.2009.121

[bib23] Hanahan D, Weinberg RA (2011) Hallmarks of cancer: the next generation. Cell 144(5): 646–674. 21376230 10.1016/j.cell.2011.02.013

[bib24] Huang, da W, Sherman BT, Lempicki RA (2009) Systematic and integrative analysis of large gene lists using DAVID bioinformatics resources. Nat Protoc 4(1): 44–57. 19131956 10.1038/nprot.2008.211

[bib25] Jiborn T, Abrahamson M, Gadaleanu V, Lundwall A, Bjartell A (2006) Aberrant expression of cystatin C in prostate cancer is associated with neuroendocrine differentiation. BJU Int 98(1): 189–196. 16831167 10.1111/j.1464-410X.2006.06345.x

[bib26] Le L, Chi K, Tyldesley S, Flibotte S, Diamond DL, Kuzyk MA, Sadar MD (2005) Identification of serum amyloid a as a biomarker to distinguish prostate cancer patients with bone lesions. Clin Chem 51(4): 695–707. 15695329 10.1373/clinchem.2004.041087

[bib27] Love HD, Booton SE, Boone BE, Breyer JP, Koyama T, Revelo MP, Shappell SB, Smith JR, Hayward SW (2009) Androgen regulated genes in human prostate xenografts in mice: relation to BPH and prostate cancer. PLoS One 4(12): e8384. 20027305 10.1371/journal.pone.0008384 PMC2793011

[bib28] Meuillet E, Stratton S, Prasad Cherukuri D, Goulet A-C, Kagey J, Porterfield B, Nelson MA (2004) Chemoprevention of prostate cancer with selenium: an update on current clinical trials and preclinical findings. J Cell Biochem 91(3): 443–458. 14755676 10.1002/jcb.10728

[bib29] Nam S, Kim D, Cheng JQ, Zhang S, Lee JH, Buettner R, Mirosevich J, Lee FY, Jove R (2005) Action of the Src family kinase inhibitor, dasatinib (BMS-354825), on human prostate cancer cells. Cancer Res 65(20): 9185–9189. 16230377 10.1158/0008-5472.CAN-05-1731

[bib30] Neill MG, Fleshner NE (2006) An update on chemoprevention strategies in prostate cancer for 2006. Curr Opin Urol 16(3): 132–137. 16679848 10.1097/01.mou.0000193388.31727.d2

[bib31] Platz EA, Helzlsouer KJ (2001) Selenium, zinc, and prostate cancer. Epidemiol Rev 23(1): 93–101. 11588860 10.1093/oxfordjournals.epirev.a000801

[bib32] Rehman I, Evans CA, Glen A, Cross SS, Eaton CL, Down J, Pesce G, Phillips JT, Yen OS, Thalmann GN, Wright PC, Hamdy FC (2012) iTRAQ identification of candidate serum biomarkers associated with metastatic progression of human prostate cancer. PLoS One 7(2): e30885. 22355332 10.1371/journal.pone.0030885 PMC3280251

[bib33] Savas S, Briollais L, Ibrahim-zada I, Jarjanazi H, Choi YH, Musquera M, Fleshner N, Venkateswaran V, Ozcelik H (2010) A whole-genome SNP association study of NCI60 cell line panel indicates a role of Ca2+ signaling in selenium resistance. PLoS One 5(9): e12601. 20830292 10.1371/journal.pone.0012601 PMC2935366

[bib34] Tafforeau L, Zorbas C, Langhendries JL, Mullineux ST, Stamatopoulou V, Mullier R, Wacheul L, Lafontaine DL (2013) The complexity of human ribosome biogenesis revealed by systematic nucleolar screening of Pre-rRNA processing factors. Mol Cell 51(4): 539–551. 23973377 10.1016/j.molcel.2013.08.011

[bib35] Tonack S, Jenkinson C, Cox T, Elliott V, Jenkins RE, Kitteringham NR, Greenhalf W, Shaw V, Michalski CW, Friess H, Neoptolemos JP, Costello E (2013) iTRAQ reveals candidate pancreatic cancer serum biomarkers: influence of obstructive jaundice on their performance. Br J Cancer 108(9): 1846–1853. 23579209 10.1038/bjc.2013.150 PMC3658525

[bib36] Uhlén M, Fagerberg L, Hallström BM, Lindskog C, Oksvold P, Mardinoglu A, Sivertsson Å, Kampf C, Sjöstedt E, Asplund A, Olsson I, Edlund K, Lundberg E, Navani S, CA-K Szigyarto, Odeberg J, Djureinovic D, Takanen JO, Hober S, Alm T, Edqvist P-H, Berling H, Tegel H, Mulder J, Rockberg J, Nilsson P, Schwenk JM, Hamsten M, von Feilitzen K, Forsberg M, Persson L, Johansson F, Zwahlen M, von Heijne G, Nielsen J, Pontén F (2015) Tissue-based map of the human proteome. Science (New York, NY) 347: 6220. 10.1126/science.1260419 25613900

[bib37] van Sluis M, McStay B (2014) Ribosome biogenesis: Achilles heel of cancer? Genes Cancer 5(5-6): 152–153. 25061498 10.18632/genesandcancer.14 PMC4104764

[bib38] Vizcaino JA, Cote RG, Csordas A, Dianes JA, Fabregat A, Foster JM, Griss J, Alpi E, Birim M, Contell J, O'Kelly G, Schoenegger A, Ovelleiro D, Perez-Riverol Y, Reisinger F, Rios D, Wang R, Hermjakob H (2013) The PRoteomics IDEntifications (PRIDE) database and associated tools: status in 2013. Nucleic Acids Res 41(Database issue): D1063–D1069. 23203882 10.1093/nar/gks1262 PMC3531176

[bib39] Vizcaino JA, Deutsch EW, Wang R, Csordas A, Reisinger F, Rios D, Dianes JA, Sun Z, Farrah T, Bandeira N, Binz PA, Xenarios I, Eisenacher M, Mayer G, Gatto L, Campos A, Chalkley RJ, Kraus HJ, Albar JP, Martinez-Bartolome S, Apweiler R, Omenn GS, Martens L, Jones AR, Hermjakob H (2014) ProteomeXchange provides globally coordinated proteomics data submission and dissemination. Nat Biotechnol 32(3): 223–226. 24727771 10.1038/nbt.2839 PMC3986813

[bib40] Wang R, Fabregat A, Rios D, Ovelleiro D, Foster JM, Cote RG, Griss J, Csordas A, Perez-Riverol Y, Reisinger F, Hermjakob H, Martens L, Vizcaino JA (2012) PRIDE Inspector: a tool to visualize and validate MS proteomics data. Nat Biotechnol 30(2): 135–137. 22318026 10.1038/nbt.2112 PMC3277942

[bib41] Wegiel B, Jiborn T, Abrahamson M, Helczynski L, Otterbein L, Persson JL, Bjartell A (2009) Cystatin C is downregulated in prostate cancer and modulates invasion of prostate cancer cells via MAPK/Erk and androgen receptor pathways. PLoS One 4(11): e7953. 19956729 10.1371/journal.pone.0007953 PMC2776515

[bib42] Welch HG, Albertsen PC (2009) Prostate cancer diagnosis and treatment after the introduction of prostate-specific antigen screening: 1986-2005. J Natl Cancer Inst 101(19): 1325–1329. 19720969 10.1093/jnci/djp278 PMC2758309

[bib43] Wheeler DL, Iida M, Dunn EF (2009) The role of Src in solid tumors. Oncologist 14(7): 667–678. 19581523 10.1634/theoncologist.2009-0009 PMC3303596

[bib44] Woodrum D, York L (1998) Two-year stability of free and total PSA in frozen serum samples. Urology 52(2): 247–251. 9697789 10.1016/s0090-4295(98)00156-3

[bib45] Yocum AK, Yu K, Oe T, Blair IA (2005) Effect of immunoaffinity depletion of human serum during proteomic investigations. J Proteome Res 4(5): 1722–1731. 16212426 10.1021/pr0501721

[bib46] Zeidan B, Cutress R, Hastie C, Mirnezami A, Packham G, Townsend P (2009a) SELDI-TOF MS proteomics in breast cancer. Clin Proteom 5(3-4): 133–147.

[bib47] Zeidan BA, Cutress RI, Murray N, Coulton GR, Hastie C, Packham G, Townsend PA (2009b) Proteomic analysis of archival breast cancer serum. Cancer Genomics Proteomics 6(3): 141–147. 19487543

[bib48] Zeidan BA, Townsend PA (2008) SELDI-TOF proteomic profiling of breast carcinomas identifies clinicopathologically relevant groups of patients similar to previously defined clusters from cDNA expression. Breast Cancer Res 10(3): 107. 18644101 10.1186/bcr2107 PMC2481502

