# Supplementary material for: Detection of candidate biomarkers of prostate cancer progression in serum: a depletion-free 3D LC/MS quantitative proteomics pilot study
Source: Br J Cancer. 2016 Sep 29;115(9):1078–86. doi: 10.1038/bjc.2016.291 (PMC5117786; doi:10.1038/bjc.2016.291)
Supplement: Supplementary Information [file bjc2016291x1.docx]

***Supplementary Methods: LC-MS Proteomics***

***Size Exclusion Chromatography (SEC) and Dialysis***

*All off-line chromatographic separations were performed on the Dionex P680 pump equipped with PDA-100 photodiode array detector. For each serum sample, a volume of 70 μL was thawed from -80°C, directly mixed with 430 μL SEC eluent composed of 6 M guanidine hydrochloride (Sigma). The mixture was subjected to Size Exclusion Chromatography using two serially connected Shodex SEC KW-804 (8.0×300 mm) columns applying an isocratic elution method at 200 μL/min. Four MW-dependent SEC protein fractions (assigned as Segments 01-04) for each specimen pool were collected.*

*The protein fractions were purified using Slyde-a-Lyzer dialysis cassettes 3 kDa MWCO rinsed into 2 L ultra-pure water renewed twice after overnight equilibrations. The purified samples were transferred in 15 mL tubes and dried with a speedvac concentrator. The residual protein content was dissolved in 100 μL triethylammonium bicarbonate 0.5 M, 0.05% SDS (dissolution buffer) with 10 min heating at 90°C and probe sonication. Protein concentration in each collected SEC fraction was measured with the Bradford assay according to manufacturer's instructions (BioRad protein assay).*

***In-solution Proteolysis and iTRAQ labelling***

*A total of 100 μg of protein from each sample derived from the same SEC segment diluted in 20 μL of dissolution buffer, were in parallel reduced by the addition of 2 μL tris-2-carboxymethyl phosphine (ΤCEP) 50 mM for 60 min at 60 °C. The cysteine residues were blocked by the addition of 1 μL methyl methanethiosulfonate (MMTS) 200 mM for 10 min at room temperature. The samples were diluted with 14 μL H_2_O prior to the addition of 6 μL trypsin (Roche) solution 500 ng/μL and were kept in dark for overnight digestion. For the iTRAQ 8-plex peptide labelling each reagent was diluted with 50 μL isopropanol and then mixed with each one of the tryptic digests. The isobaric tags used for the samples are detailed in Figure 1. Samples (8) were pooled after 2 h of reaction and the mixture was dried with a speedvac.*

***Hydrophylic interaction liquid chromatography (HILIC)***

*First dimension peptide chromatographic separation was based on hydrophilic interaction chromatography using the core-shell technology Kinetex HILIC column (150×2.1 mm, 2.6 µM). Mobile phase A was composed of 100% acetonitrile, 0.1% formic acid and mobile phase B was composed of 100% H_2_O, 0.1% formic acid. Samples were dissolved in 20 μL 1:1 mixture of mobile phase A and B and were subjected to multi-step gradient separation as follows: For 5 min isocratic 10% B, for 87 min gradient up to 31% B, for 20 min gradient up to 51%, for 18 min gradient up to 95% B, for 60 min isocratic 95% B and for 5 min gradient down to 5% B. Flow rate was 65 μL/min and column temperature was set at 35°C. Fractions were collected in a peak dependent manner based on 280 nm signal response, dried with speedvac concentrator and stored at -20°C until the LC-MS analysis.*

***LC-MS analysis***

*The LC-MS experiments were performed on the Dionex Ultimate 3000 UHPLC system coupled with the high resolution nano ESI Orbitrap Elite mass spectrometer (Thermo Scientific). Each peptide fraction was reconstituted in 25 μL loading solution (2% acetonitrile, 0.1% formic acid) and a 4 μL volume was loaded on the Acclaim PepMap 100, 100 μm × 2 cm C18, 5 µM, 100 Ȧ trapping column with the ulPickUp Injection mode. The loading pump flow rate was 5 μL/min and samples were loaded on the trapping column for 8 min. For the low pH peptide separation the Acclaim PepMap RSLC, 75 μm × 25 cm, nanoViper, C18, 2 μm, 100 Ȧ column retrofitted to a distal coated emitter (FS360-20-10-D-20-C7) was used for multi-step gradient elution. Mobile phase A was composed of 2% acetonitrile, 0.1% formic acid and mobile phase B was composed of 100% acetonitrile, 0.1% formic acid. The gradient elution method at flow rate 300 nL/min and column temperature set at 35°C was as follows: for 70 min up to 40% B, for 5 min up to 85% B, for 5 min 85% B, for 2 min re-equilibration to 3% B, for 8 min isocratic 3% B.*

*Gaseous phase transition of the peptides was performed with positive ion electrospray ionisation applying a voltage of 2.5 kV. Top 7 multiply charged precursors within 350 -1900 m/z and intensity threshold 500 counts were selected with FT mass resolution of 120,000 and isolated for both CID and HCD fragmentation within mass windows of 2 and 1.2 Da respectively. Normalised collision energy was set at 35 for the CID spectra and 40 for the HCD spectra that were acquired with FT resolution of 15,000 within a m/z range of 100-1900. Already targeted precursors were dynamically excluded for further isolation and activation for 30 sec with 10 ppm mass tolerance.*

***Protein Identification and Quantification***

*All tandem mass spectra were submitted to the Sequest search engine implemented on the Proteome Discoverer software version 1.3.0.339 for peptide and protein identification. All spectra were searched against an updated UniProt Fasta file containing 20,218 human reviewed entries (downloaded May 2014).*

*The Sequest node for the HCD spectra included the following parameters: Precursor Mass Tolerance 10 ppm, Fragment Mass Tolerance 20 mmu, Dynamic Modifications were Oxidation of M (+15.995 Da), Deamidation of N, Q (+0.984 Da), Phosphorylation of S (+79.966 Da) and Static Modifications were iTRAQ8plex at any N-Terminus, K, Y (+304.205 Da) and Methylthio at C (+45.988 Da). The Fragment Mass Tolerance for the CID spectra was 0.5 Da while search for phosphorylation was not included. The level of confidence for peptide identifications was estimated using the Percolator algorithm with decoy database searching. Strict FDR was set to 0.01, relaxed FDR was set to 0.05 and validation was based on q-Value. The Reporter Ion Quantifier node included a custom iTRAQ 8plex (Thermo Scientific Instruments) Quantification Method, integration window tolerance 20 ppm and integration method Most Confident Centroid. Protein ratios were normalised to protein median and phosphorylation localization probability was estimated with the phosphoRS node. Only uniquely occurring peptides were used for protein relative quantification and peptides with missing iTRAQ channels were excluded.*

***Supplementary Methods: ELISA validation***

*Briefly, standards were prepared and samples were diluted as determined by an optimisation step performed previously, then 100 µL of these were added to the appropriate wells of the ELISA plate and incubated at 37°C for 2 hours. Following aspiration of the samples and standards, 100 µL of biotin conjugated antibody was added and incubated for a further 1 hour at 37°C. Wells were washed with the provided Wash Buffer and then HRP-avidin detection reagent added and incubated at 37°C for 30 minutes (USCN kits) or 1 hour (Cusabio kits and VWA5B2 My Biosource kit). Detection reagent was then removed and the wells washed again with Wash Buffer before addition of substrate solution and incubation for a further 15-30 minutes at 37°C. A stop solution was then added and the optical density of each well read at 450 nm.*

*The TSR1 My Biosource kit procedure was different in that 50 µL standard and sample were used and were added to the plate followed by the addition of 100 µL of HRP-conjugate followed by a 60 minute incubation at 37°C. After four washes with the wash buffer, 50 µL each of Chromogen solution A and 50 µL of Chromogen solution B were added to the wells and incubated at 37°C for 15 minutes. Finally, 50 µL of stop solution was added prior to the plate being read at 450 nm.*

***Supplementary Methods: Literature and Network Analysis***

*Using Biorelate’s in house ‘Bespoke database (DB)’ platform, relevant publications were assigned to each of the 7 markers (including orthologues), using all full texts in open access PMC and all abstracts in PubMed (2015 version of Medline). These were then further categorised as PCa related and PCa biomarker related. Fisher’s exact test was performed to calculate the enrichment of each marker’s association with PCa study and PCa biomarker study, utilising the hypergeometric mean as follows: (a) PCa related / PCa biomarker related publications for that marker (b) all other publications that were PCa related / PCa biomarker related (c) other publications for that marker that were not PCa related / PCa biomarker related (d) all other publications that were related to any disease aside from PCa.*

$$\rho=\frac{\frac{a+b}{a} \times\frac{c+d}{c}}{\frac{a+b+c+d}{a+c}}$$

Equation S1. Fisher’s exact test using the hypergeometric mean

*Proteins found to interact both directly and indirectly with the markers (not including KLK3), were then extracted from their associated publications using Bespoke DB and manually validated by a biomedical expert to ensure their accuracy. Speculative interactions (hypothesised or suggested links) were not included. Due to the large amount of manual validation required to review the data extracted for KLK3, only direct interactions sourced from STRING (*[*Szklarczyk et al, 2015*](file:///\\filestore.soton.ac.uk\users\sl4v08\mydocuments\personal\paper\MS-paper-v1%2041response.docx#_ENREF_35)*) were used in subsequent analyses.*

*Additional direct interactions of proteins not extracted from the published literature for the other six genes were also included from STRING. A network of the markers and proteins interacting with at least two markers was created using Cytoscape 3.2.1.(*[*Shannon et al, 2003*](file:///\\filestore.soton.ac.uk\users\sl4v08\mydocuments\personal\paper\MS-paper-v1%2041response.docx#_ENREF_34)*). Edges are colour coded by type and both direct and indirect interactions are included in each network. Arrowheads are used to indicate network directionality. DAVID biological process and molecular function GO term enrichment analysis was performed using the proteins that formed the connected graph.*
