# Supplementary material for: Detection of candidate biomarkers of prostate cancer progression in serum: a depletion-free 3D LC/MS quantitative proteomics pilot study
Source: Br J Cancer. 2016 Sep 29;115(9):1078–86. doi: 10.1038/bjc.2016.291 (PMC5117786; doi:10.1038/bjc.2016.291)
Supplement: Supplementary Table 1 [file bjc2016291x2.docx]

| Table S1: ELISA kit catalogue numbers and the companies they were purchased from. | | |
| --- | --- | --- |
| Marker | **Catalogue number** | **Company** |
| KLK3 | ABIN414938 | Antibodies Online |
| CST3 | ABIN414432 | Antibodies Online |
| SGCd | ABIN419868 | Antibodies Online |
| SRC | ABIN824036 | Antibodies Online |
| SAA | ABIN414457 | Antibodies Online |
| VWA5B2 (cohort 1) | MBS9343383 | My Biosource |
| VWA5B2 (cohort 2) | ABIN855348 | Antibodies Online |
| TSR1 (cohort 1) | MBS281756 | My Biosource |
| TSR1 (cohort 2) | ABIN825379 | Antibodies Online |
