# Supplementary figures and images for: Detection of candidate biomarkers of prostate cancer progression in serum: a depletion-free 3D LC/MS quantitative proteomics pilot study
Source: Br J Cancer. 2016 Sep 29;115(9):1078–86. doi: 10.1038/bjc.2016.291 (PMC5117786; doi:10.1038/bjc.2016.291)

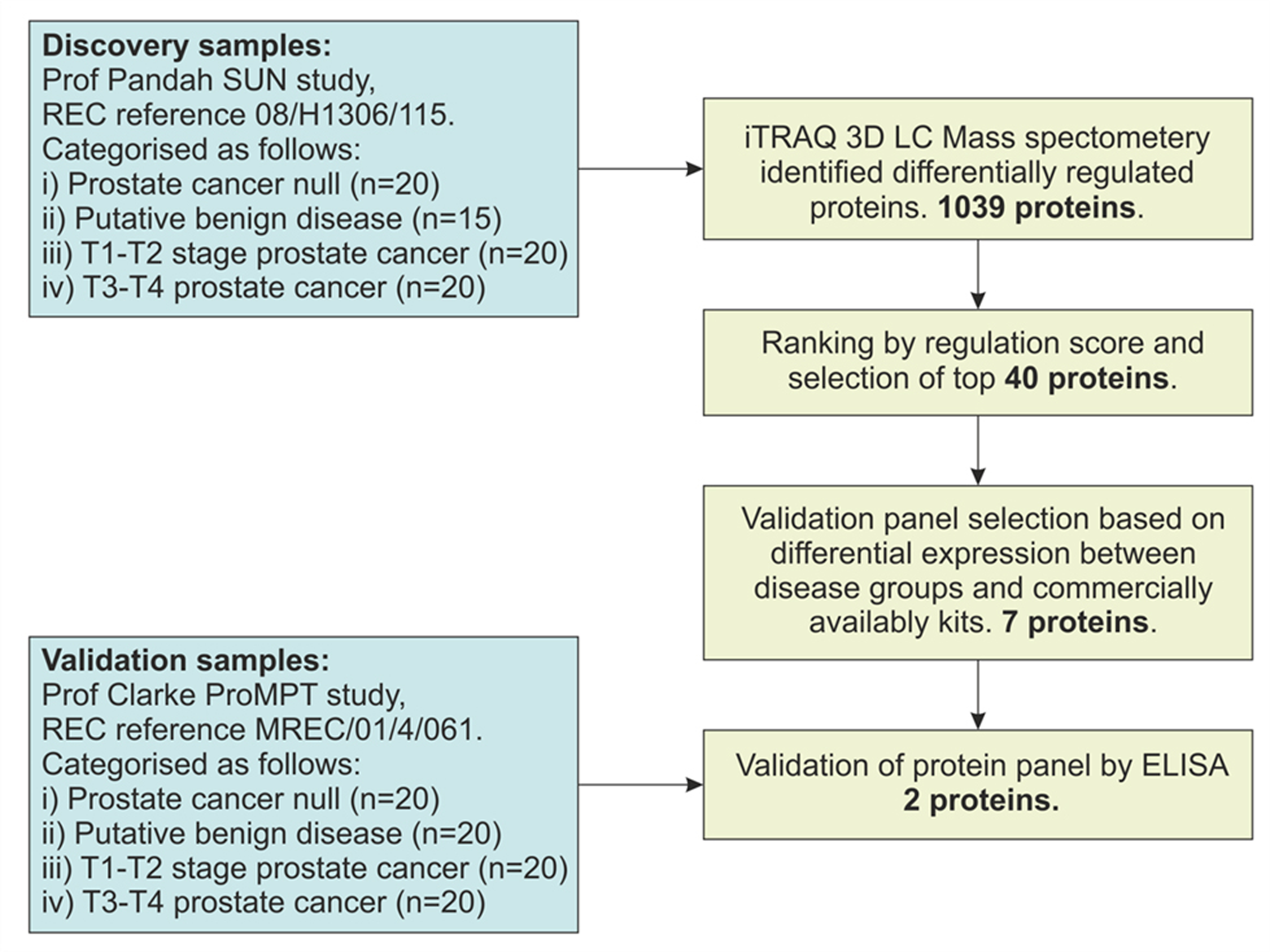

Supplement: Supplementary Figure 1 [file bjc2016291x5.tif]

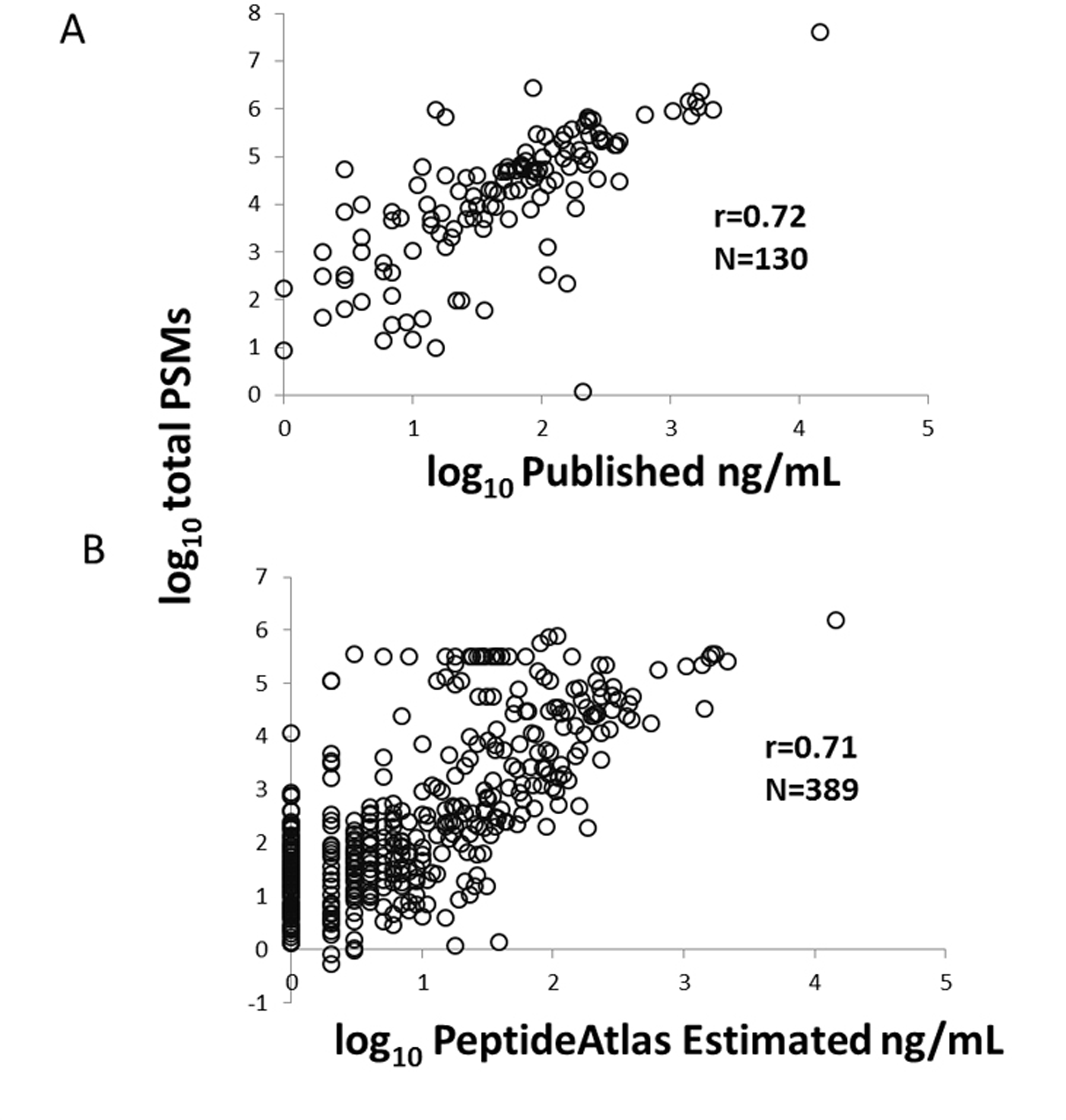

Supplement: Supplementary Figure 2 [file bjc2016291x6.tif]

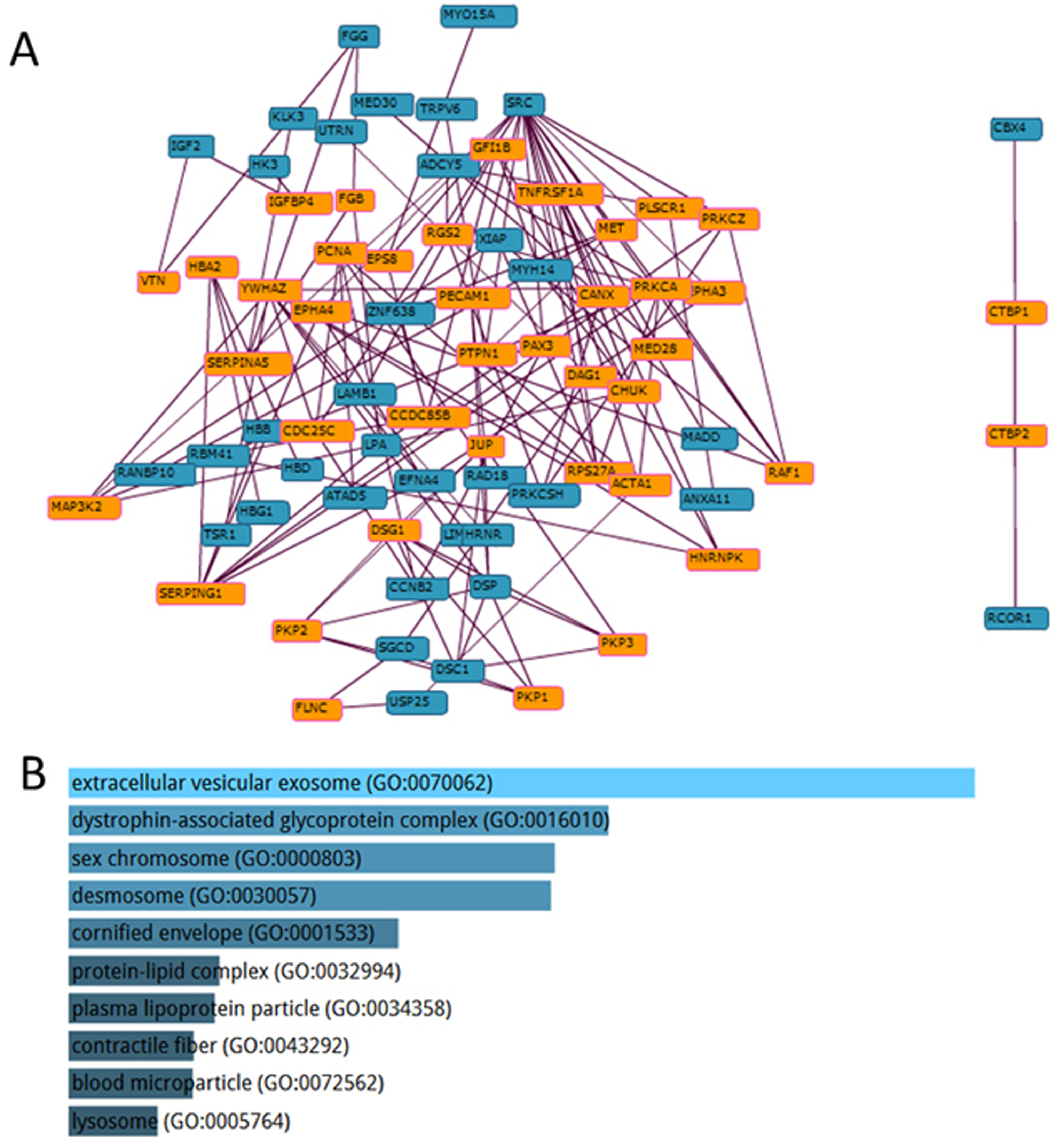

Supplement: Supplementary Figure 3 [file bjc2016291x7.tif]
